# Non-invasive Brain Stimulation for Chronic Pain: State of the Art and Future Directions

**DOI:** 10.3389/fnmol.2022.888716

**Published:** 2022-05-26

**Authors:** Huan-Yu Xiong, Jie-Jiao Zheng, Xue-Qiang Wang

**Affiliations:** ^1^Department of Sport Rehabilitation, Shanghai University of Sport, Shanghai, China; ^2^Huadong Hospital, Shanghai, China; ^3^Department of Rehabilitation Medicine, Shanghai Shangti Orthopaedic Hospital, Shanghai, China

**Keywords:** non-invasive brain stimulation, chronic pain, neuromodulation, tDCS, rTMS

## Abstract

As a technique that can guide brain plasticity, non-invasive brain stimulation (NIBS) has the potential to improve the treatment of chronic pain (CP) because it can interfere with ongoing brain neural activity to regulate specific neural networks related to pain management. Treatments of CP with various forms of NIBS, such as repetitive transcranial magnetic stimulation (rTMS) and transcranial direct current stimulation (tDCS), using new parameters of stimulation have achieved encouraging results. Evidence of moderate quality indicates that high-frequency rTMS of the primary motor cortex has a clear effect on neuropathic pain (NP) and fibromyalgia. However, evidence on its effectiveness regarding pain relief in other CP conditions is conflicting. Concerning tDCS, evidence of low quality supports its benefit for CP treatment. However, evidence suggesting that it exerts a small treatment effect on NP and headaches is also conflicting. In this paper, we describe the underlying principles behind these commonly used stimulation techniques; and summarize the results of randomized controlled trials, systematic reviews, and meta-analyses. Future research should focus on a better evaluation of the short-term and long-term effectiveness of all NIBS techniques and whether they decrease healthcare use, as well as on the refinement of selection criteria.

## Introduction

Conceptually, chronic pain (CP) is a process of neuroplasticity disorder caused by excitatory and inhibitory imbalances in pain processing pathways (Peyron and Fauchon, 2019). According to the United States Centers for Disease Control and Prevention, the prevalence of CP in the general population in 2018 is between 11 and 40%, and it costs the economy between $560 billion and $635 billion annually in the United States alone (Steglitz et al., 2012; Dahlhamer et al., 2018). A systematic review of 19 studies conducted in the United Kingdom showed that the mean prevalence of CP is 43.5% (Fayaz et al., 2016). Approximately 40% of patients with CP report difficulty in pain control, and over 60% reveal inadequate pain relief from medications (Breivik et al., 2006). CP is the dynamic result of a range of biological, psychological, and social factors, and the most common medications no longer provide sufficient analgesia for most patients. Therefore, specific, predictable, and effective modalities of CP management must be explored and developed.

## Using Basic Scientific Knowledge to Develop an Effective, Neural Circuit-Based Treatment for Chronic Pain

**FIGURE 1:**
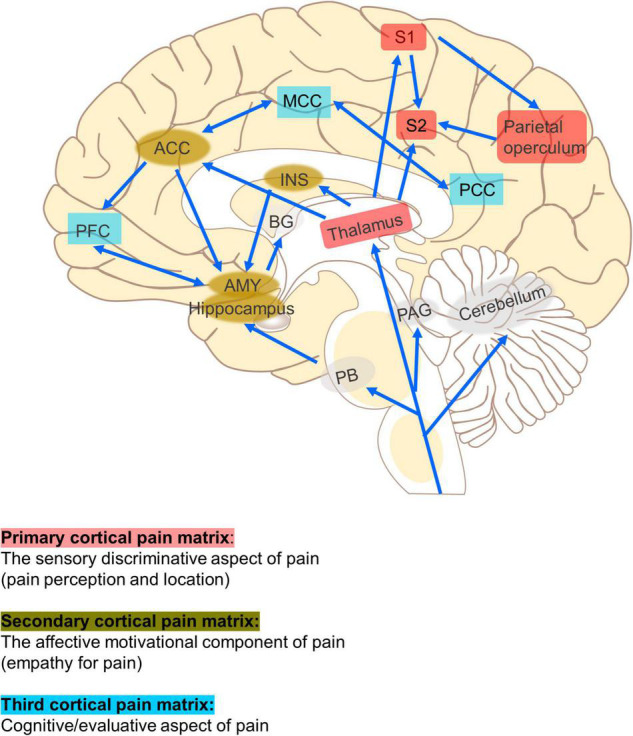
Neural networks related to pain. The primary cortical pain matrix (thalamus, S1, S2, posterior insula, and parietal operculum) contributes to pain perception and location. The secondary (ACC, INS, AMY, and hippocampus) represent common structures identified in the affective motivational pain pathway, such as empathy for pain. The third (PFC, MCC, and PCC) represents one component of the cognitive evaluative pain system. The arrows represent multiple cortical connections between regions and systems indicating the complex interconnectedness of brain regions involved with pain. ACC, anterior cingulate cortex; PFC, prefrontal cortex; AMY, amygdala; PAG, periaqueductal gray; RVM, rostroventral medulla; DH, dorsal horn; INS, insula; S1, primary somatosensory cortex; S2, secondary somatosensory cortex; MCC, medial cingulate cortex; PCC, posterior cingulate cortex; PB, parabrachial nucleus.

Non-invasive brain stimulation (NIBS) has the potential to improve the treatment of CP because it can regulate specific neural networks related to pain processing in the brain, such as the anterior cingulate cortex (ACC) and the thalamus, and promote a downward pain suppression mechanism to relieve pain (Peyron et al., 2007; Figure 1). Imaging studies in humans have suggested that CP is the result of changes in neural networks and central pain mechanisms, including perception, sensitization, and pain regulation pathways (Perocheau et al., 2014). Moreover, pain is a personal experience that requires different brain circuits to process. Therefore, NIBS technique may be effective in relieving pain by exciting or inhibiting specific neural networks associated with pain processing.

**FIGURE 2:**
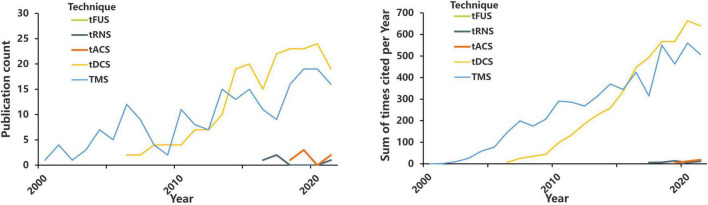
The number of annual publications and annual citations on TMS, tDCS, tACS, tRNS, tFUS, and pain. https://www.webofscience.com/wos/alldb/basic-search; search dates from 2000 to 2021.

As an alternative intervention to invasive brain stimulation, NIBS can remarkably reduce the incidence of invasive stimulation. Over the past 20 years, the number of studies that used NIBS techniques for pain treatment has exponentially grown, a trend that possibly reflects the importance of this field (Figure 2 and Table 1). Thus far, NIBS has been applied to manage various CP conditions, including fibromyalgia (Forogh et al., 2021), neuropathic pain (NP) (Galhardoni et al., 2019), and migraine (Schading et al., 2021). Although the neural mechanisms underlying the analgesic effects of NIBS are not yet fully understood, the mechanisms behind the functional effects of each NIBS technique are different (Leocani et al., 2019). Moreover, the analgesic effects of NIBS seem to be highly correlated with the stimulated brain regions. Therefore, we hypothesize that patients with different types of CP may benefit from stimulation of different target brain regions through different NIBS techniques. At the cellular level, stimulation can change the electrical state of individual neurons; at the neurohumoral signal level, stimulation can cause neurotransmitter activity; at the network level, stimulation can change neuronal circuits; at the behavioral level, stimulation can cause changes in pain and function (Knotkova et al., 2021).

**TABLE 1:** Systematic and evidence-based reviews of non-invasive brain stimulation techniques for chronic pain.

	Sample size	Disease	Intervention	Duration of the trial period	Follow-up	Primary outcomes	Main results	AMSTAR-2 rating
**rTMS**								
Zhang et al., 2021	29 studies (24 for rTMS) 826 participants (736 for rTMS)	Neuropathic pain	Intervention: rTMS Control: sham stimulation	1 session (minimum)–15 sessions (maximum)	NA	Pain level (VAS and NRS)	rTMS successfully relieved the pain symptoms of 715 (97.1%) NP patients. The stimulation parameters of rTMS that best induce an analgesic effect are a target cortex of M1, a stimulation frequency of 10–20 Hz, 1000–2000 pulses, and 5–10 sessions.	Low
Jin et al., 2015	32 studies 589 participants	Neuropathic pain	Intervention: HF-rTMS (>1 Hz) over the M1 Control: sham stimulation, parallel-group, and crossover designs	1 session–10 sessions	1 month (minimum)–2 months (maximum)	Pain level (VAS and NRS)	All 3 HF-rTMS treatments (5, 10, and 20 Hz) produced pain reduction, while there were no differences between them, with the maximal pain reduction found after 1 and 5 sessions of rTMS. This significant analgesic effect remained for 1 month after 5 sessions of rTMS treatment.	Moderate
Saltychev and Laimi, 2017	7 RCTs 210 participants	Fibromyalgia	Intervention: rTMS Control: sham stimulation, other treatment, or no control treatment	8 sessions–24 sessions	1 week–3 months	Pain level (VAS and NRS)	Both pooled results of pain severity (−1.2 for NRS and −0.7 for VAS) were below the minimal clinically important difference of 1.5 points. There is moderate evidence that rTMS is not more effective than sham in reducing the severity of pain in fibromyalgia patients.	Moderate
Knijnik et al., 2016	5 RCTs 143 participants	Fibromyalgia	Intervention: rTMS Control: sham stimulation	10 sessions–14 sessions	NA	Pain intensity (VAS and BPI) Depression (HDRS and BDI) QoL (FIQ)	In comparison with sham stimulation, rTMS demonstrated a superior effect on the QoL of patients with FM 1 month after starting therapy. rTMS showed a trend toward reducing pain intensity but did not change depressive symptoms.	Moderate
Stilling et al., 2019	34 studies (6 for TMS, 16 for rTMS) 631 participants	Headache (19/22 studies for migraine)	Intervention: TMS and rTMS Control: sham stimulation or alternative standard of care (i.e., botulinum toxin, headache drug)	1 session–23 sessions	4 weeks–20 weeks	Headache frequency, duration, intensity, use of abortive medications, depression, anxiety, and QoL.	There is moderate evidence for rTMS contributes to reductions in headache frequency, duration, intensity, abortive medication use, depression, and functional impairment. However, only a few studies reported changes greater than sham treatment.	Low
Lan et al., 2017	5 RCTs 313 participants	Migraine	Intervention: rTMS Control: sham stimulation	1 session–23 sessions	NA	Pain intensity	Single-pulse transcranial magnetic stimulation is effective for the acute treatment of migraine with aura after the first attack (*p* = 0.02). The efficacy of TMS on chronic migraine was not significant (*p* = 0.14).	High
**tDCS**								
Zhang et al., 2021	29 studies (5 for tDCS) 826 participants (95 for tDCS)	Neuropathic pain	Intervention: tDCS Control: sham stimulation	1 session–10 sessions	NA	Pain level (VAS and NRS)	Five studies involving 95 NP patients (76.0%) showed that tDCS successfully relieved NP. The most effective parameters of tDCS are a current intensity of 2 mA, a session duration of 20–30 min, and 5–10 sessions.	Low
David et al., 2018	8 studies 127 participants	Neuropathic pain	Intervention: tDCS Control: sham stimulation	1 session–20 sessions	90 min–6 months	Pain level (VAS and NRS)	All of the studies showed significant effects of tDCS on NP (spinal cord injury, stroke, and amputation) when compared to the control group, except for one with SCI and another related to radiculopathy. Positive effects in the follow-up studies lasted up to 7 days.	Low
Mehta et al., 2015	5 studies 83 participants	Neuropathic pain after spinal cord injury	Intervention: tDCS Control: sham stimulation	1 session–10 sessions	NA	Pain level (VAS and NRS)	The pooled analysis found a significant effect of tDCS on reducing neuropathic pain after SCI post-treatment (*p* = 0.012); however, this effect was not maintained at follow-up (*p* = 0.194).	Moderate
Lloyd et al., 2020	14 studies 452 participants	Fibromyalgia	Intervention: tDCS Control: sham stimulation	1 session–10 sessions	NA	Pain level (VAS and NRS)	Meta-analysis of data from 8 controlled trials provides tentative evidence of pain reduction when active tDCS is delivered compared to sham.	
Zhu et al., 2017	6 studies 192 participants	Fibromyalgia	Intervention: tDCS Control: sham stimulation alone or sham stimulation combined with other interventions for fibromyalgia	1 session–10 sessions	NA	Pain level (VAS and NRS)	Significant improvement in pain and general fibromyalgia related function was seen with anodal transcranial direct current stimulation over the primary motor cortex (*p* < 0.05). However, the pressure pain threshold did not improve (*p* > 0.05).	High
Stilling et al., 2019	34 studies (12 for tDCS) 413 participants	Headache (8/12 studies for migraine)	Intervention: tDCS Control: sham stimulation or alternative standard of care (i.e., botulinum toxin, headache drug)	5 session–20 sessions	NA	Headache frequency, duration, intensity, use of abortive medications, depression, anxiety, and QoL.	There is moderate evidence for tDCS in the treatment of headaches concerning reduction in headache frequency, intensity, abortive medication use, depression, and anxiety.	Low
Feng et al., 2019	9 studies (4 for tDCS) 276 participants (115 for tDCS)	Migraine	Intervention: tDCS Control: sham stimulation or no control treatment	10 session–20 sessions	1 month–12 weeks	Pain level (VAS and NRS)	tDCS over the M1 or the DLPFC showed significant effects on reducing headache intensity in patients with migraine.	Moderate
Alwardat et al., 2020	9 studies 411 participants	Chronic low back pain	Intervention: tDCS Control: sham stimulation	1 session–15 sessions	3 weeks–6 months	Pain level (VAS and NRS)	The meta-analysis showed non-significant effect of multiple sessions of tDCS over M1 on pain reduction (*p* = 0.249) and disability (*p* = 0.434) post-treatment, respectively.	Low

*Reviews selected to avoid redundancy with text and other reviews. AMSTAR-2 ratings represent a grading system for systematic reviews, with confidence in findings rated as high, moderate, low, or critically low based on 16 items (13 for reviews without meta-analyses). AMSTAR, A Measurement Tool to Assess Systematic Reviews; NIBS, non-invasive brain stimulation; RCTs, randomized controlled trials; TMS, transcranial magnetic stimulation; sTMS, single-pulse TMS; dTMS, deep transcranial magnetic stimulation; rTMS, repetitive transcranial magnetic stimulation; HF-rTMS, high frequency transcranial magnetic stimulation; tDCS, transcranial direct current stimulation; VAS, Visual Analog Scale; QoL, quality of life; NP, neuropathic pain; SCI, spinal cord injury; RMT, resting motor threshold; BPI, brief pain inventory; HDRS, Hamilton Depression Rating Scale; BDI, beck depression inventory; NRS, Numeric Rating Scale; MPQ, McGill Pain Questionnaire.*

**FIGURE 3:**
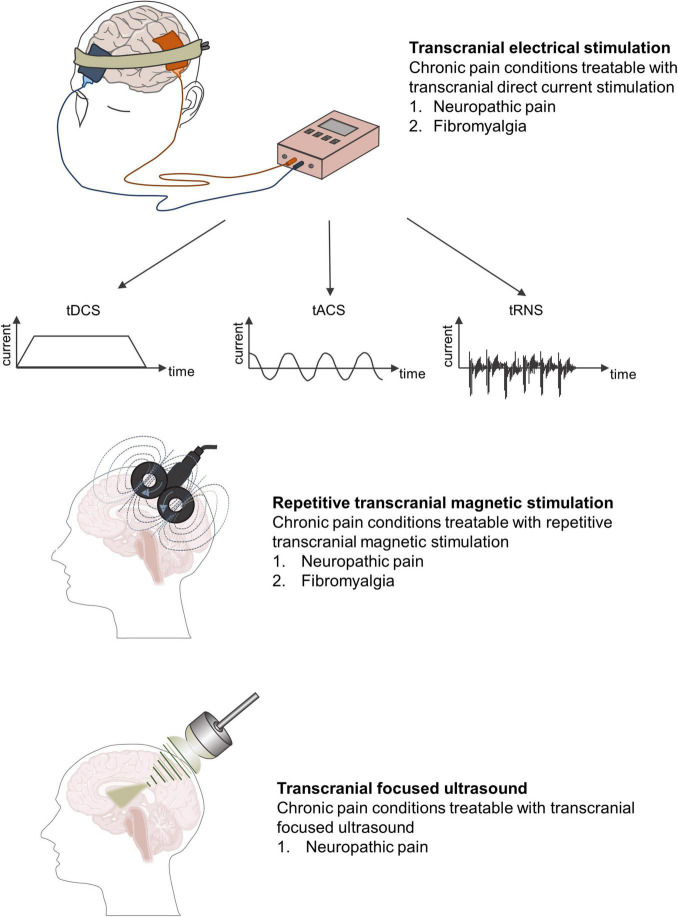
Different forms of non-invasive brain stimulation techniques and chronic pain conditions most amenable to treatment.

Given that most NIBS techniques modulate neural activity in a frequency- or polar-dependent manner, most applications of CP research have involved excitatory NIBS, such as high-frequency transcranial magnetic stimulation (TMS) or anodal transcranial direct current stimulation (tDCS) (Figure 3). In this review, we outline the possibilities and limitations of the NIBS approach in CP research, with particular emphasis on best practices and selected developments.

## Repetitive Transcranial Magnetic Stimulation

### Underlying Neurophysiological Mechanisms of Repetitive Transcranial Magnetic Stimulation in Chronic Pain Treatment

Transcranial magnetic stimulation uses dynamic magnetic fields to generate induced currents to regulate individual neurons and neuron groups in the cortex and the neural networks connected to them (Young et al., 2014). Strong effects can sufficiently depolarize neurons to trigger action potentials (Young et al., 2014). If TMS pulse stimulation is repeatedly given, it is called repetitive TMS (rTMS).

#### Regulating the Excitability of the Pain Loop

In general, the stimulatory effect of rTMS depends on the frequency: high-frequency stimulation (≥5 Hz) increases cortical excitability, whereas low-frequency stimulation (≤1 Hz) decreases it (Hoogendam et al., 2010). The excitability induced by high-frequency rTMS (HF-rTMS) may be the result of the weakened intracortical inhibition mediated by the gamma-aminobutyric acid (GABA) rather than directly caused by increased excitability (Ziemann, 2004). By contrast, low-frequency rTMS (LF-rTMS) may enhance GABA-mediated intracortical inhibition, thereby reducing cortical excitability.

The rationale for applying rTMS to treat pain is that it can modulate neural activity in cortical and subcortical brain structures associated with pain processing, such as the thalamus, in both local and remote brain regions. Compared with electrical stimulation, magnetic stimulation allows the study of local nerve tissue activation, where the signal is not hindered by other tissues and is minimally invasive to humans. In turn, the activation of the cortical structure transmits the action potential to neural circuits related to pain processing, such as the cingulate cortex and the thalamus, regardless if it is forward or reverse (Strafella et al., 2001; Perocheau et al., 2014). Previous studies have confirmed that rTMS can also directly excite the thalamus through the cortical–thalamic projection system, thereby inhibiting the transmission of injury information through the spinothalamic pathway (Bestmann et al., 2004; Martin et al., 2013). In other words, when HF-rTMS is used, the pain information transmitted through the spinothalamic tract and the ipsilateral thalamic nucleus may be suppressed. By contrast, when LF-rTMS is utilized, pain transmission may be unsuppressed.

#### Synaptic Plasticity

**FIGURE 4:**
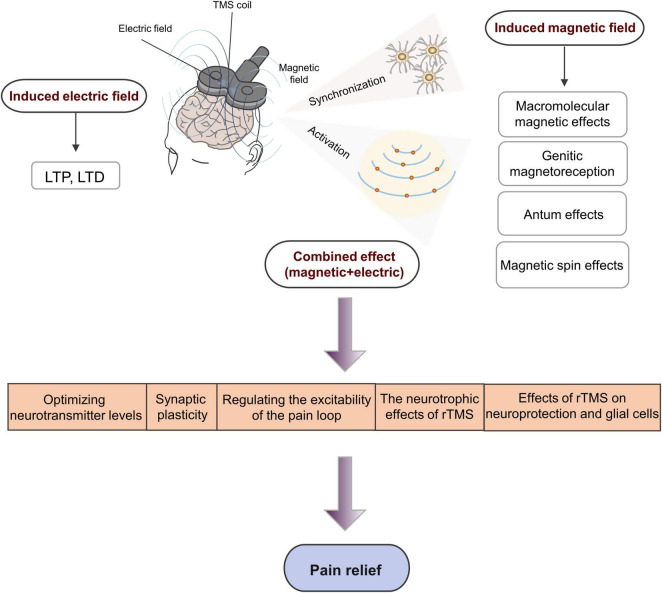
Schematic diagram demonstrating the underlying neurophysiological mechanism of rTMS involved in pain management. LTP, long-term potentiation; LTD, long-term depression.

Another mechanism by which TMS alleviates pain is by changing the plasticity of the nervous system, whereas long-term changes in neuron excitability are associated with long-term changes in synaptic effects, especially long-term potentiation (LTP) and long-term depression (LTD). Similar to basic synaptic physiology, enhancing the synaptic strength is often referred to as LTP, whereas reducing the synaptic strength is referred to as LTD (Figure 4).

Repetitive transcranial magnetic stimulation exerts an accumulation effect through repeated, continuous, and regular stimulation that can excite more neurons. More importantly, rTMS can affect the brain functions of local and remote areas and realize the reconstruction of cortical functions (Moisset et al., 2016). Pridmore et al. (2005) reported that a single session of LF-rTMS reduced the excitability of the primary motor cortex (M1) region for about 15–30 min. However, after multiple and continuous sessions of LF-rTMS, the excitability of the M1 region decreased, which lasted for 30 min on the first day and extended to 2 h on the second day. Therefore, rTMS can probably cause cumulative plasticity changes in brain neural tissues. A few words should be added regarding the mechanisms of analgesic action of rTMS delivered to M1. A previous study highlighted a significant release of endogenous opioids within a bihemispheric brain network involved in the perception and modulation of pain, which was produced by a single session of 10 Hz rTMS of M1 in a positron emission tomography (PET) study based on 10 healthy subjects (Lamusuo et al., 2017). This was consistent with previous observations made in CP patients treated with invasive epidural motor cortex stimulation (Maarrawi et al., 2007, 2013). However, the mechanisms of action of M1 stimulation in pain are surely more complex and multiple, involving various pain modulatory systems concerned with emotion, attention, and/or sensory discrimination processing, related to various neural pathways connecting different brain regions, thalamic nuclei, and/or the spine, and with various neurotransmitter systems beyond endogenous opioids, such as glutamate, GABA, and/or dopamine for example (Nguyen et al., 2011; Lefaucheur, 2016; Moisset and Lefaucheur, 2019). All of these factors can contribute to the development of long-term synaptic plasticity that provides significant pain relief beyond the time of stimulation.

#### Optimizing Neurotransmitter Levels

Many studies have proved that the analgesic mechanism of rTMS is not only due to the induction of LTP or LTD in the process of neuron depolarization and hyperpolarization, either of which leads to changes in nerve excitability and synaptic connections, but also due to secondary changes in neurotransmitter secretion related to pain. Studies have reported that the release of endogenous opioids in the ACC and periaqueductal gray (PAG) matter is related to the noxious effects of rTMS (de Andrade et al., 2011). rTMS can optimize neurotransmitter levels, promote the release of endogenous opioids and the secretion of brain-derived neurotrophic factors, and increase the concentration of GABA, thereby improving pain (Dall’Agnol et al., 2014). Lefaucheur et al. (2006) found that HF-rTMS of M1 can reduce pain and enhance cortical inhibition, and the degree of pain reduction is positively correlated with intracortical inhibition. They speculated that rTMS may regulate the balance between inhibitory neurotransmitters and excitatory glutamate neurotransmitters in the cerebral cortex, thus achieving an analgesic effect.

#### Improving Regional Cerebral Blood Flow and Metabolism in the Brain

Notably, changes in regional cerebral blood flow (rCBF) and metabolism after rTMS treatment may be correlated with the decrease in pain score. Tamura et al. (2004) found that after LF-rTMS treatment, the rCBF of the medial prefrontal cortex was remarkably reduced, and the rCBF of the tail of the ACC and the contralateral premotor area was considerably increased. Meanwhile, the subjects’ pain was substantially reduced. In addition, the degree of pain relief was positively correlated with the decrease in rCBF in the medial prefrontal cortex, suggesting that the analgesic effect caused by rTMS is related to changes in rCBF (Tamura et al., 2004).

### Repetitive Transcranial Magnetic Stimulation for Neuropathic Pain

The pathophysiological mechanism of NP may be related to changes in the structural or functional plasticity of the central nervous system (Leung et al., 2009). The maintenance of NP mainly depends on central sensitization. Central sensitization refers to the abnormal increase in excitability or synaptic transmission of central pain-related neurons, including the increase in spontaneous discharge activity of neurons, the expansion of sensory domains, and the reduction of the threshold value to external stimuli, thus amplifying the transmission of pain signals (Nickel et al., 2012). rTMS acts on the cerebral cortex and adjacent structures under the cortex through high-level regulation of the central nervous system, and it exerts various effects on the pain process, thereby exerting an analgesic effect. Yang et al. (2018) suggested that HF-rTMS may reduce central sensitization and relieve NP by downregulating the overexpression of neuronal nitric oxide synthase in ipsilateral dorsal root ganglions and inhibiting the activity and proliferation of astrocytes in the L4–L6 spinal dorsal horns ipsilateral to NP.

A meta-analysis of 25 studies (589 long-term follow-up patients) evaluated the efficacy of HF-rTMS for the treatment of NP (Jin et al., 2015). Pooled analgesic results showed a statistically significant effect size of −0.86 (*p* < 0.05), indicating that rTMS can effectively reduce the pain intensity of NP from different sources. The result indicated that a single rTMS treatment can remarkably reduce the pain intensity of patients with NP. When the number of sessions was increased from 2 to 10, the subjects also produced considerable pain relief, especially among patients with pain after suffering from central stroke. After 1–2 months of follow-up (161 participants), the analgesic effects of multiple rTMS treatments (≥5 sessions) were observed to last at least 1 month but not more than 2 months, indicating that the different analgesic effects of rTMS may depend on the neuroanatomical source of the pathophysia of NP, that is, the more effective source of the therapeutic effects of rTMS on NP is the “top” (supraspinal, cranial, or spinal) rather than the “bottom” (nerve roots or peripheral nerves). Similarly, Zhang et al. (2021) systematically reviewed 29 studies (24 for rTMS, 736 participants), and found that rTMS successfully improved the pain symptoms of 97.1% of patients with NP (715 participants). The analgesic effect of rTMS was maintained for 2 weeks after the last session, but this beneficial effect usually lasted for less than 1 month. In addition, the M1 region was targeted in almost all patients (82.5%, 607 participants).

As recent reviews have concluded, the application of HF-rTMS of M1 may alleviate various types of NP. Some of the NP conditions with the greatest response to rTMS include post-stroke central pain and trigeminal neuralgia, whereas NP conditions with more peripheral anatomical origins, such as post-traumatic peripheral NP, are less reactive to rTMS (Lefaucheur, 2016; Leung et al., 2020). Although a few studies suggest this conclusion, we think it is premature to present this type of assertion which is not based on published data on large series. It is unclear whether any particular type of NP would be considered a better indication for rTMS treatment.

### Repetitive Transcranial Magnetic Stimulation for Fibromyalgia

Fibromyalgia is usually insensitive to conventional treatment. Previous studies indicated that rTMS may work by modulating pain pathways (Bestmann et al., 2004; Yoo et al., 2008), such as the descending inhibitory pathway, and by modulating social–affective areas of the brain, such as the right temporal lobe (Boyer et al., 2014). A meta-analysis (7 studies, 210 patients) evaluated the efficacy of HF-rTMS of M1 in the treatment of fibromyalgia (Saltychev and Laimi, 2017). Based on the patients’ scores on a 0–10 numerical rating scale, their pain intensity before and after the last rTMS session decreased by 1.2 points. Moreover, the pain intensity before the last stimulation and 1 week to 1 month after the last stimulation decreased by 0.7 points. Both pooled results were statistically significant but below the cut-off point of a minimum clinically significant difference of 1.5 points. In a narrative review that analyzed 12 studies on fibromyalgia, 9 studies concluded that rTMS of M1 was effective in relieving pain in patients with this condition (Yang and Chang, 2020). By contrast, the remaining three randomized controlled trials (RCTs) denied that patients with fibromyalgia benefited from rTMS.

Although the results of some studies were negative, the fact that fibromyalgia is difficult to manage suggests that rTMS is a potential analgesic method for managing fibromyalgia.

### Repetitive Transcranial Magnetic Stimulation for Migraine

Recent studies suggested that the mechanism of migraine may be linked to neurological causes, such as cerebral cell hyperexcitability and altered cortical excitability (Cosentino et al., 2014; Lan et al., 2017). On the one hand, rTMS can regulate the excitability of cortical structures involved in pain control, such as inhibiting cortical spreading depression (Chervyakov et al., 2015). Therefore, rTMS may contribute to the prevention and relief of headache symptoms during migraine attacks. On the other hand, rTMS can promote the release of endogenous analgesic substances, such as dopamine and endogenous opioid peptides, thereby relieving headaches.

Currently, in the United States, acute migraine with aura is the only FDA-approved indication for single-pulse TMS45. A meta-analysis also reported that single-pulse TMS was effective in the acute treatment of aura migraine after the first attack (*p* = 0.02), but not in chronic migraine (*p* = 0.14) (Lan et al., 2017). However, another systematic review found that TMS and rTMS contributed to reductions in headache frequency, duration, intensity, abortive medication use, depression, and dysfunction in both chronic primary and secondary headaches (Stilling et al., 2019). In addition, consistent with the findings of Lan et al. (2017), only a few studies reported greater changes than sham stimulation (Stilling et al., 2019). Similarly, another systematic review found that, compared with sham stimulation, the outcome indicators of patients suffering from migraine who received HF-rTMS treatment substantially improved, including headache frequency, pain intensity, headache duration, and dosage (Yang and Chang, 2020). However, two studies showed that the results after rTMS were not superior to those after sham stimulation, and both showed a strong placebo response. Despite conflicting evidence on its efficacy, rTMS may still be a potential option for patients with migraine.

### Repetitive Transcranial Magnetic Stimulation for Chronic Low Back Pain

Previous studies have demonstrated that abnormal postural control of trunk muscles may lead to the occurrence of chronic low back pain (CLBP) (Deliagina et al., 2006). Furthermore, the M1 region is believed to play a key role in postural control regulation (Ambriz-Tututi et al., 2016). Ambriz-Tututi et al. (2016) evaluated the long-term effects of rTMS on CLBP and compared them with those of physical therapy and sham stimulation. Results showed that the patients who received HF-rTMS of M1 had a remarkable decrease in pain intensity after 1 week of treatment. After 3 weeks of continuous treatment, their pain intensity was reduced by nearly 80% compared with the baseline and was substantially lower than that of the group that received sham stimulation or physical therapy. In addition, a previous study reported that, compared with sham stimulation, 1 session of HF-rTMS can significantly lessen the pain intensity felt by patients with CLBP (*p* < 0.001) (Johnson et al., 2006). Compared with those on fibromyalgia and headache, there is less evidence to suggest the efficacy of rTMS on CLBP.

### Summary of Repetitive Transcranial Magnetic Stimulation

In Canada, Australia, Japan, the European Union, and Israel, TMS devices have been approved for depression, schizophrenia, and NP (Marangell et al., 2007). However, in the United States, the application of TMS to pain management is only considered investigational.

Thus far, most high-quality RCTs and systematic reviews have shown that rTMS applied to the dorsolateral prefrontal cortex (DLPFC), the supplementary motor area (SMA), or the primary somatosensory cortex (S1) lacks analgesic effects, while stimulation of M1 provides pain relief (Marangell et al., 2007; Lefaucheur et al., 2020). In 2020, the guidelines for the use of rTMS developed by an expert panel in Europe stated that HF-rTMS of M1 has a clear effect on NP and fibromyalgia (level A evidence) (Lefaucheur et al., 2020). The selection of stimulation targets and stimulation parameters is the most basic and important issue that determines the analgesic effect of rTMS. Some of the current issues in stimulating targets are as follows: (1) whether different types of CP conditions require specific stimulation targets; (2) whether combined treatment with different stimulation targets will enhance their analgesic effect; (3) lack of evidence from studies that compared the analgesic effects of different stimulation targets; and (4) few use image-guided navigation to improve the accuracy and repeatability of targeting. Another potential concern with rTMS is the duration effect. The number of sessions in most TMS studies ranged from 1 to 10 sessions. Some studies have reported that the analgesic effects of rTMS are cumulative and require multiple sessions to achieve clinically significant effects (Brighina et al., 2009). A single rTMS session may not be sufficient to induce changes in cortical excitability, and multiple sessions are required to induce changes in neuroplasticity. However, prolonged use of rTMS to increase stimulation intensity may also lead to a reduction or even reversal of the stimulatory effect of motor cortical excitability, making the treatment counterproductive. Therefore, the number of sessions with rTMS should be investigated to provide best practice for the analgesic effects of rTMS.

## Transcranial Direct Current Stimulation

### Underlying Neurophysiological Mechanisms by Which Transcranial Direct Current Stimulation Relieves Chronic Pain

Transcranial direct current stimulation works by using two or more electrodes to apply a low-amplitude direct current (typically from 0.5 to 2 mA) to specific brain regions to modulate their excitability, thereby relieving pain (Nitsche and Paulus, 2000). Traditional tDCS usually uses two sponge electrodes, one as the anode and the other as the cathode. HD tDCS utilizes a set of smaller electrodes, such as 4 × 1, to provide more focused stimulation.

#### Selective Excitability of Neurons

**FIGURE 5:**
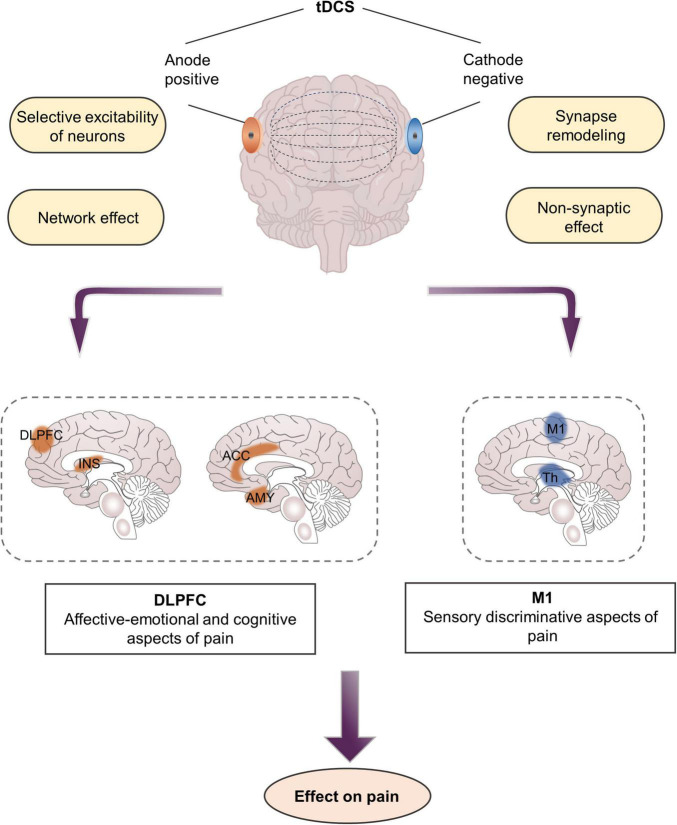
Mechanisms and targets of transcranial direct current stimulation in pain management. DLPFC, dorsolateral prefrontal cortex; M1, primary motor cortex; INS, Insula; Th, thalamus.

Unlike TMS, tDCS employs a weak current and generally does not cause an action potential but only changes the resting membrane potential of nerve cells, thereby regulating the excitability of nerve cells (Nitsche et al., 2008). The change in membrane potential is the physiological basis of the immediate regulatory effect of tDCS. The effect of tDCS on cortical excitability depends on polarity: anodal stimulation leads to depolarization of the nerve membrane, thereby increasing excitability, whereas cathodal stimulation results in hyperpolarization of the nerve membrane, which in turn inhibits excitability (Lefaucheur et al., 2017; Lefaucheur and Wendling, 2019; Figure 5). However, changes in cell membrane potential do not explain the subsequent effects of cessation of tDCS, such as the persistence of analgesic effects several weeks after stimulation.

Notably, the cortical excitation effect of tDCS is related to the direction and intensity of the current, but it is not a linear relationship, that is, the greater the current intensity, the better the stimulus effect (Utz et al., 2010). Sometimes this effect will be reversed with the increase in current intensity. The immediate to long-term effects of tDCS vary depending on the selected stimulation parameters (Shekhawat et al., 2016). Studies have reported that the analgesic effect of tDCS is cumulative, requiring multiple sessions to achieve clinically significant results (Lefaucheur et al., 2017). In general, a stimulation lasting for at least 5 min is needed to produce biological effects. Changes in neural activity occur not only during tDCS but also several hours after stimulation has ended.

#### Network Effect

Aside from regulating local activity at the stimulus site, tDCS also exerts network effects that alter structural and functional connections between different brain regions (Cummiford et al., 2016; Lin et al., 2017). A PET study of patients with CP showed that motor cortex stimulation acts as a “gate” that triggers the activity in the stimulated and distant brain tissues, including the thalamus, anterior insula, and PAG (Garcia-Larrea and Peyron, 2007). Specifically, anodal stimulation of the M1 region alleviates pain by activating various neural circuits in the precentral gyrus, which may be afferent or efferent to structures connected to the sensory or emotional components of pain processing, such as the thalamus or ACC (Lefaucheur, 2006; Nguyen et al., 2011). Cummiford et al. (2016) found that, compared with sham stimulation, applying 20 min of anodal tDCS over left M1 five times can reduce functional connectivity among the left ventrolateral thalamus and the medial prefrontal lobe, and the left auxiliary motor area, as well as functional connectivity among the right ventrolateral thalamus and the cerebellum and the left auxiliary motor area in patients with fibromyalgia. These brain regions are the components of the pain matrix involved in the processing and regulation of pain, especially the emotional components of pain (Figure 1).

#### Synapse Remodeling

Aside from changing the polarity of membrane potential, tDCS also regulates the synaptic microenvironment and modulates neuronal function at the synaptic level. tDCS interacts with several neurotransmitters, including serotonin, dopamine, and acetylcholine, and affects various neuronal membrane channels, such as sodium and calcium ions (Lefaucheur et al., 2017). In general, direct current stimulates cortical neurons to regulate the expression of NMDA receptors and the release of GABA, resulting in LTP or LTD, both of which cause synaptic remodeling (Stagg and Nitsche, 2011). Previous studies have argued that calcium-dependent synaptic plasticity in glutamate neurons plays a key role in the mechanism of long-lasting neuroplasticity in tDCS because blocking NMDA receptors attenuates the after-effects of tDCS. In addition, tDCS can alleviate pain by modulating the thalamic inhibitory network and interfering with the cortex–cortical and cortex–subcortical synaptic connections related to pain formation (Liebetanz et al., 2002; Batsikadze et al., 2013).

#### Non-synaptic Effect

Although tDCS regulates resting membrane potentials at the synaptic level, it more commonly regulates resting membrane potentials along the entire axon, which may lead to non-synaptic effects (Ardolino et al., 2005). These non-synaptic mechanisms of tDCS are probably due to conformational and functional changes in various axon molecules. When exposed to a direct current electric field, various phenomena, such as transmembrane ion conductance, membrane structure changes, cytoskeleton changes, or axon transmission, will occur around axons (Jefferys, 1995). In addition, Zheng et al. (2011) found that the effect of tDCS is related to changes in cerebral blood flow. After they administered cathodal tDCS, the blood flow substantially decreased and lasted for a period of time. This condition may also be the key mechanism underlying the analgesic effect of tDCS.

### Transcranial Direct Current Stimulation for Neuropathic Pain

In addition to network effects and changes in central nervous excitability, tDCS may also alleviate NP by regulating the central nervous immune system and inhibiting glial cell activation. Cioato et al. (2016) investigated the effects of tDCS on nociceptive responses and measured IL-1 β, IL-10, and TNF-α levels in the central nervous system structure of NP rats. After tDCS, the levels of IL-1β and TNF-α decreased, whereas those of IL-10 increased, suggesting that tDCS may modulate the immune system to alleviate NP. Recent studies have established that microglia and astrocytes in the nervous system play key roles in the initiation and maintenance of NP, respectively (Gritsch et al., 2016). As a common method for regulating cortical excitability in superficial pain-related areas, tDCS can inhibit neuronal sensitivity after peripheral nerve injury and downregulate the expression of the P2 × 4 receptor, thereby inhibiting microglia activity, ultimately leading to NP remission (Zhang et al., 2020).

A systematic review (8 studies, 127 participants) suggested that, compared with sham stimulation, tDCS could notably reduce pain intensity in patients with NP associated with spinal cord injury (SCI), stroke, and amputation, and its analgesic effect lasted for 1 week after the end of the intervention (David et al., 2018). However, no significant differences between the groups were observed in patients with radiculopathy. Similarly, a meta-analysis reported a moderate effect of tDCS in reducing NP in patients with SCI; however, the effect was not maintained at follow-up (Mehta et al., 2015). A mean pooled decrease of 1.33 units on a 10-item scale was found post treatment. In another systematic review (6 studies, 125 patients with NP), 5 studies found that, compared with sham stimulation, anodal tDCS remarkably alleviated NP (Zhang et al., 2021). Overall, many studies have confirmed that tDCS has a moderate effect on pain relief among individuals with chronic NP, but this effect is not maintained during follow-up.

### Transcranial Direct Current Stimulation for Fibromyalgia

The pathogenesis of fibromyalgia may be related to the dysfunction of the central nervous system (Cook et al., 2007; Brown et al., 2014). As a neuromodulation technique that targets the central nervous system, tDCS may theoretically help relieve pain. A meta-analysis of 6 studies (192 patients with fibromyalgia) reported that, compared with sham stimulation, anodal tDCS of M1 was more likely to relieve pain and improve fibromyalgia-related function, whereas cathodal tDCS of M1 and anodal tDCS of left DLPFC did not produce notable analgesic effects (Zhu et al., 2017). Although this meta-analysis was unable to calculate the overall effect of tDCS on fibromyalgia during the follow-up period, some of the studies it included concluded that 10 sessions of anodal tDCS over M1 was more likely to control pain than 5 sessions, and this effect might last up to 2 months. Similarly, Lloyd et al. (2020) also reported that active tDCS applied at an intensity of 2 mA to left M1 for 20 min/days for 10 sessions appears to be able to lower pain intensity in fibromyalgia. Hou et al. (2016) reviewed 16 studies (5 for tDCS, 11 for rTMS; 572 patients with fibromyalgia), found that aside from improving cognitive function, both tDCS and rTMS had similar positive effects on pain symptoms, sleep disturbances, and tender spots. However, rTMS produced a greater analgesic effect than tDCS. Therefore, excitatory rTMS/tDCS should be considered in the treatment of patients with fibromyalgia, especially for those with painful symptoms that are not responding to other therapies or for whom the continuation of such therapies is not possible due to their adverse side effects (as is commonly the case with FDA-approved drugs).

### Transcranial Direct Current Stimulation for Migraine

A systematic review of 12 studies on chronic headache (8 for migraine, 413 participants) found that, compared with baseline, tDCS substantially reduced headache frequency in 7 studies (Stilling et al., 2019). However, only one study showed that, compared with sham stimulation, tDCS considerably decreased headache frequency. Six studies reported that tDCS shortened headache duration, but only one study was statistically different from the control group. Seven studies found that tDCS reduced pain intensity, but only two studies showed significant differences between groups. Similarly, Feng et al. (2019) reviewed 9 studies (4 for tDCS, 115 participants), and found that anodal tDCS of M1 markedly reduced the frequency and intensity of headaches in patients suffering from migraine. Moreover, tDCS over DLPFC substantially reduced pain intensity in these patients, but it had no notable effect on attack frequency. In addition, compared with sham stimulation, cathodal tDCS applied to the vertex or visual cortex did not remarkably change the frequency and intensity of headaches in these patients. Compared with those on fibromyalgia and NP, there is less evidence to suggest the efficacy of tDCS on migraine.

### Transcranial Direct Current Stimulation for Chronic Low Back Pain

Unlike acute low back pain, non-specific CLBP usually has no peripheral cause (Latremoliere and Woolf, 2009). Central mechanisms have been hypothesized to explain the development and maintenance of pain. Great functional connectivity between the dorsal medial PFC–amygdala–accumbens circuit in patients with subacute low back pain contributes to the risk of CP (Vachon-Presseau et al., 2016). Therefore, brain network disturbance is considered one of the possible causes of CLBP. A recent systematic analysis (eight studies) revealed that, compared with sham stimulation, 1 session of tDCS treatment resulted in substantial pain relief (Patricio et al., 2021). By contrast, multiple sessions of tDCS treatment did not improve short-term and medium-term pain. In 2019, based on two studies on tDCS, an expert panel proposed a level A recommendation against the use of tDCS of M1 for the treatment of CLBP (Baptista et al., 2019).

Recent systematic analyses did not support the use of tDCS for CLBP treatment, and evidence of low quality suggests that tDCS negatively affects CLBP. A possible explanation for these negative findings is that the pathogenesis of CLBP is affected by multiple factors. Therefore, the participants enrolled in these reviews might have had other mechanical diseases or complications, such as cervical spondylosis or small joint disorders. In addition, visceral pain involving the lower back can be a misleading condition, leading doctors to misdiagnose or miss a diagnosis.

### Summary of Transcranial Direct Current Stimulation

In the United States, tDCS has not been approved for any clinical indications but is only used as a research technique for pain management. The guidelines for neurostimulation therapy of CP issued by the European Academy of Neurology gave “weak recommendations” for the use of tDCS for the treatment of peripheral NP and “uncertain recommendations” for the treatment of fibromyalgia (Cruccu et al., 2016; Knotkova et al., 2021). In the United States, tDCS has not been approved for any clinical indications but is only used as a research technique for pain management. The effectiveness of tDCS in relieving CP may vary according to pain subtype, including spontaneous, paroxysmal, and persistent pain (Soler et al., 2010). Thus far, many high-quality RCTs and systematic reviews have shown that the application of tDCS to DLPFC, SMA, or S1 lacks analgesic effects, whereas stimulation of the M1 region provides pain relief (Lefaucheur et al., 2017). It is worth noting that tDCS is non-local with a network effect, and cortices adjacent to the stimulation target may also be affected. As a result, it is essential to combine tDCS with neuroimaging and functional connectivity analysis so that we can attribute specific efficacy to neuromodulation of M1 only, but unfortunately, the majority of tDCS studies lack the corresponding neuroimaging evaluation. In addition, the current level of evidence supporting the positive effect of the application of tDCS over M1 on pain relief is considerably lower than that of rTMS. Compared with that of rTMS, the after-effect of tDCS is also less obvious (Ngernyam et al., 2013).

## Transcranial Alternating Current Stimulation

Transcranial alternating current stimulation (tACS) changes the nerve oscillation signal by applying a sinusoidal alternating current stimulation with a fixed amplitude and frequency to the brain, thereby regulating pain intensity (Antal and Herrmann, 2016; Tavakoli and Yun, 2017; Arendsen et al., 2018). Previous studies have confirmed that the neural oscillation signals in the alpha and gamma bands before pain stimulation can regulate the individual’s perception of pain stimulation (Tu et al., 2016). Given that the neural synchronization effect is related to endogenous neural oscillation signals, the stimulation frequency selected in analgesia studies is the frequency corresponding to the neural oscillation signals closely related to pain processing, such as the alpha and gamma neural oscillation signals (Tu et al., 2016). After the pain stimulation, the neural oscillation signals in the alpha band are weakened, whereas the nerve oscillation signals in the gamma band are strengthened. The neural oscillation signal in the gamma band is not affected by the saliency of the stimulus but reflects the individual’s perception of pain intensity, and it can encode intra- and interindividual pain sensitivity (Zhang et al., 2012; Hu and Iannetti, 2019). Thus, the use of tACS in regulating pain perception has a theoretical basis.

So far, only one study has investigated the analgesic effect of tACS on fibromyalgia. An RCT of 15 patients with fibromyalgia found that tACS combined with physical therapy administered 5 days per week for 2 weeks effectively reduced pain Visual Analog Scale (VAS) scores immediately after the intervention (Bernardi et al., 2021). However, this positive analgesic effect was no longer present 4 weeks after the end of the intervention. Similarly, only one study has investigated the effectiveness of tACS for CLBP treatment. Ahn et al. (2019) found that applying tACS with an amplitude of 1 mA and a frequency of 10 Hz to the F3 and F4 electrodes of EEG electrode caps for 40 min can reduce the pain intensity of patients with CLBP. Moreover, tACS enhances the alpha oscillation signal intensity of the electrode near the somatosensory area, and this increase in the alpha oscillation signal is also strongly related to the decrease in pain intensity.

Despite its appeal, tACS seems to be rarely used in the field of CP research. Apart from somatosensory areas, few studies have evaluated the analgesic effect of tACS on CP in other brain areas (Ahn et al., 2019; Bernardi et al., 2021). Although previous studies have demonstrated that the application of alpha tACS over S1 can enhance alpha oscillations and thus induce pain relief, the quality of evidence is extremely low.

## Transcranial Random Noise Stimulation

As an innovative form of electrical stimulation, transcranial random noise stimulation (tRNS) is based on the principle of stochastic resonance that uses alternating current with a frequency randomly varying between 0 and 640 Hz to increase the excitability of the cortex regardless of the orientation of the current (Paulus, 2011). Studies have shown that weak tRNS of M1 led to enhanced motor cortical excitability, where high-frequency subdivision of the whole tRNS spectrum between 100 and 640 Hz was functionally responsible for inducing excitability enhancement (Terney et al., 2008; Paulus, 2011; Moret et al., 2019). In addition, 10 min of tRNS stimulation was reported to induce a consistent excitability increase lasting over 1 h after stimulation (Terney et al., 2008). This effect could be attributed to the repeated opening of sodium channels or to the increased sensitivity of neuronal networks to field modulation (Terney et al., 2008; Paulus, 2011). These evidence raise the possibility that tRNS may prove as an effective and reliable means to relieve pain perception.

Curatolo et al. (2017) applied 10 sessions of tRNS of M1 to 20 women with fibromyalgia. The results showed that, compared with placebo, active tRNS remarkably reduced pain and fibromyalgia impact questionnaire scores. By contrast, Palm et al. (2016) reported that, immediately after tRNS, no notable intergroup differences in mean pain VAS score, attention performance, and mood scale were observed between the tRNS and placebo groups.

Evidence supporting the effects of tRNS as a single intervention for CP treatment is limited. Moreover, drawing conclusions on whether tRNS is useful in this situation is difficult. Therefore, large multicenter RCTs are warranted to evaluate the better potential of tRNS for pain management.

## Transcranial Focused Ultrasound

As a NIBS method that can focus on deep brain structures, transcranial focused ultrasound (tFUS) can stimulate deep brain targets with a high level of spatial resolution and generate superimposed ultrasonic pulses deep in the brain by using transducers containing piezoelectric elements (Aubry and Tanter, 2016; Folloni et al., 2019). Through this feature, tFUS can target almost any part of the peripheral or central nervous system (Xiao and Zhang, 2018). Previous studies have demonstrated that adjusting ultrasound parameters can produce different physiological effects on the nervous system, ranging from reversible activation or suppression of neural activity (low intensity, low-frequency ultrasound) to irreversible tissue ablation (high intensity focused ultrasound) (di Biase et al., 2019). tFUS can produce an analgesic effect through various mechanisms, such as by increasing blood–brain barrier permeability, improving the concentration of central acting analgesics in the central nervous system, or modulating gene expression in pain perception (di Biase et al., 2019). If the neuromodulation of tFUS can act on specific neural networks related to pain processing, it may be developed for pain management. Currently, tFUS has been approved only for thalamotomy in chronic NP and for ablation of specific tumors (di Biase et al., 2021).

In a cross-controlled study of 19 healthy adults, Badran et al. (2020) found that tFUS targeting the right anterior thalamus could modulate the antinociceptive effects of the pain processing network. Compared with sham stimulation, a 20-min tFUS treatment significantly increased the heat pain threshold; but tFUS did not remarkably alter the heat pain tolerance threshold. Their findings suggested that tFUS could modulate pain sensitivity through its interaction with the thalamus and by affecting the afferent sensory-discriminative component of pain. Compared with other NIBS techniques focusing on CP, there is less evidence to suggest the efficacy of tFUS on CP.

## Future Directions

New insights into the neuromodulation mechanisms underlying CP have opened new perspectives on new treatments. Over the past 20 years, NIBS techniques have emerged as one of the most promising tools for treating pain. NIBS may be an effective treatment for alleviating CP as indicated by many Cochrane meta-analyses of CP syndromes. However, in most cases, the corresponding analgesic effects are weak and variable. Except for rTMS, which has been proved effective in the treatment of major depression, no NIBS protocol has been certified as a routinely used CP management tool.

Given that the brain is an advanced center for pain control, CP treatment based on neural network systems may be the future trend of transcranial modulation of brain activities. On the one hand, researchers must value multi-site approaches to target various networks or sites of networks. On the other hand, researchers must study further the functions of pain-related nuclei in the brain and control CP through precise regulation of pain-related nuclei in the brain. Progress in NIBS research requires a deeper understanding of the relationship between the underlying neurophysiological effects and functional outcomes of pain, as well as better identification of clinical and non-clinical factors that influence pain reactivity.

## Author Contributions

H-YX collected the data, elaborated the design of the study, and wrote the manuscript. X-QW and J-JZ organized the research project and corrected the manuscript. All authors read and approved the manuscript.

## Conflict of Interest

The authors declare that the research was conducted in the absence of any commercial or financial relationships that could be construed as a potential conflict of interest.

## Publisher’s Note

All claims expressed in this article are solely those of the authors and do not necessarily represent those of their affiliated organizations, or those of the publisher, the editors and the reviewers. Any product that may be evaluated in this article, or claim that may be made by its manufacturer, is not guaranteed or endorsed by the publisher.
